# Supplementary material for: Causal relationship between periodontal disease-related phenotype and knee osteoarthritis: A two-sample mendelian randomization analysis
Source: PLoS One. 2024 May 31;19(5):e0304117. doi: 10.1371/journal.pone.0304117 (PMC11142551; doi:10.1371/journal.pone.0304117)
Supplement: Attachment — Submitted filename: response letter.docx [file pone.0304117.s001.docx]

**Response to Reviewers’ Comments**

We appreciate the critical reading of our manuscript entitled “*Causal Relationship Between Periodontal Disease-Related Phenotype and Knee Osteoarthritis: A Two-sample Mendelian Randomization Analysis*”, and we thank you for all the valuable suggestions received from the two reviewers. We have carefully considered the comments and have revised the manuscript accordingly. The responses to the comments are listed one by one as follows (*please see below*).

We are looking forward to hearing about your final decision.

**Gang Deng**

**E-mail address:** dgflying@126.com

*Affiliation: Institute of Blood Transfusion at Ningbo Central Blood Station, Ningbo, Zhejiang, China. 315010*

**Response to Reviewer #1**

Dear Prof. Kashoo, we greatly appreciate the time and effort you had dedicated to providing detailed and constructive feedback on our manuscript. Your insights have been invaluable in guiding our revisions to improve the clarity, accuracy, and overall quality of our work. Below, we address each of your points specifically:

**Title Concerns**

- **Comment**: The title does not reflect the study population and design. Authors need to follow standard guidelines (PICO) to frame the title of the study.
- **Response**: We appreciate the reviewer's suggestion and have revised the title to better reflect the study population and design using the PICO framework:" Causal Relationship Between Periodontal Disease-Related Phenotype and Knee Osteoarthritis: A Two-sample Mendelian Randomization Analysis ".

**General Impression from the Abstract**

- **Comment**: Concerns about the validity of instrumental variables, potential confounding factors, and the generalizability of findings.

**Response**: We appreciate the reviewer's thoughtful considerations regarding the validity of instrumental variables, potential confounding factors, and the generalizability of our findings. To address these concerns, we have conducted a meticulous screening process for instrumental variables, as detailed in the Methods section, to ensure their validity and adherence to Mendelian Randomization assumptions. Additionally, we have implemented sensitivity analyses, including the MR-Egger regression and weighted median approaches, to assess and mitigate potential pleiotropy and biases. Regarding potential confounding factors, we have introduced a multivariable Mendelian Randomization approach incorporating smoking status, alcohol consumption, and BMI as covariates to explore their impact on the observed causal relationships. In the Discussion section, we openly acknowledge the limitations and complexities inherent in MR studies and highlight the need for cautious interpretation. Furthermore, we stress the importance of considering the specific population and context when generalizing our findings. These measures collectively aim to enhance the robustness, transparency, and applicability of our study results.

- **Comment**: The study focuses on Europeans, and generalizing the findings to other populations may be limited due to genetic and environmental differences.
- **Response:** We agree with the reviewer's concern about the generalizability of our findings to non-European populations. In the Discussion section, we have acknowledged this limitation and highlighted the need for similar MR studies in diverse demographic groups to validate and potentially extend our results. We have also emphasized that genetic and environmental factors associated with periodontal disease and knee osteoarthritis may vary substantially across different ethnicities, cautioning against broad extrapolation to non-European populations.
- **Comment**: The accuracy and reliability of results depend on the quality of available GWAS data. Issues such as imputational accuracy and genotyping errors could affect outcomes.
- **Response:** We appreciate the reviewer's attention to the quality of GWAS data in our analysis. To strengthen the reliability of our instrumental variables, we have meticulously implemented a stringent screening process based on p-values and F-values, as detailed in the Methods section. Furthermore, we want to highlight the significance of our data sources, emphasizing not only the meticulous screening process but also the utilization of large-scale GWAS datasets with stringent quality control measures. In the Methods Data Sources section, we will provide additional details regarding the reliability and quality of the datasets used in our analysis and insert appropriate references to support the credibility of our chosen data sources. This will ensure a more comprehensive understanding of the robustness and validity of our results.
- **Comment**: The analysis may not account for all potential confounding factors influencing both PDRP and KOA, impacting the validity of causal inferences.
- **Response**: We included smoking, alcohol consumption, and BMI as confounding factors in multivariate Mendelian randomization analysis, and the results showed that there was no direct causal relationship between KOA and PDRP when smoking, alcohol consumption, and BMI were considered in the multivariate analysis. In addition, the MR-Egger P values in both directions were 0.706 and 0.917, respectively, suggesting that independence (genetic variation only affects outcomes through this outburst) and exclusivity (independent of confounders) were established.
- **Comment**: The study assumes a bidirectional relationship, but the causal directionality between PDRP and KOA might be more complex, involving other variables not considered in this analysis.
- Response: Screening was carried out through PhenoScanner, and independence and exclusivity were established.

**Introduction**

- **Comments and Responses**: We have addressed each specific comment as follows:

1. Clarified the first sentence to explicitly state the progressive nature of KOA.
2. Specified the high incidence of KOA within our study population using recent epidemiological data.
3. Replaced "escalating" with "increasing" for simplicity.
4. Added statistics on the socioeconomic impact of OA.
5. Clarified the reference to the increase in prevalence and its relationship with life expectancy and disability due to OA, citing relevant GBD studies.
6. Specified the global ranking of CP and corrected the typographical error ("Total").

- **Comments**: General Impression: Authors need to have the complete manuscript edited by an author proficient in English editing, as it is currently difficult to understand the article.
- **Response:** We sincerely appreciate your feedback regarding the clarity of our manuscript. We understand the importance of clear and accessible scientific communication. To address your concerns, we have completed a thorough revision and editing of the manuscript with the assistance of an author proficient in English editing. Our goal with these revisions is to ensure that the article is easier to understand and that our findings and arguments are communicated more effectively. We hope that these changes will facilitate a better understanding of our research for all readers. Thank you for bringing this to our attention and for your constructive comments.
- **The full text has been edited by native English speakers**

**Methods**

- **Comment:** The p-value was 5.00E-08. It does not make sense to me.
- **Response:** Thank you for raising this important point. The choice of a stringent p-value threshold, such as 5.00E-08, in MR studies, including GWAS, is grounded in established statistical practices within the genetics research community.The adoption of the 5.00E-08 threshold for declaring genome-wide significance is widely supported in the literature. For instance, a seminal work by Dudbridge (2013) emphasized the necessity of stringent thresholds in GWAS to control the family-wise error rate, especially when dealing with a massive number of multiple comparisons [1]. This threshold helps mitigate the risk of false-positive associations in the face of millions of genetic variants tested against traits or diseases.Moreover, the reliability of the 5.00E-08 threshold has been underscored in a comprehensive review by Evangelou and Ioannidis (2013), which discussed the challenges and solutions in genetic epidemiology, emphasizing the significance of stringent thresholds to address the issue of multiple testing [2].In the context of Mendelian Randomization, it's crucial to recognize that the strong correlation between SNPs and exposures is one of the three fundamental assumptions of MR. This assumption, often referred to as the "Relevance" assumption, posits that the genetic variants used as instrumental variables must be associated with the exposure of interest [3]. This ensures that the genetic instrument is relevant to the exposure, a critical condition for valid causal inference in MR.
- **Comment**: Moreover, the reliability and validity of data extracted from different sources are questionable.
- **Response**: Thank you for your insightful feedback on our study. Recognizing the importance of double samples from different cohorts, we want to clarify that our study meticulously adheres to this criterion. Specifically, distinct samples from separate cohorts were utilized for the PDRP and KOA datasets to enhance the robustness of our analysis. The reliability and validity of genetic data, particularly SNPs, within the same ethnic or population groups serve as foundational strengths, fostering stability across diverse datasets. We emphasize that public databases and published sources were employed, and we would like to reference seminal works [1,2] to substantiate the reliability of these data sources. Leveraging the consistency of genetic markers across diverse samples, our MR study aims to provide reliable insights into the genetic underpinnings of disease, contributing valuably to clinical and epidemiological research. We appreciate your insights, and any further suggestions are welcomed.
- **Comment**: The complete paper emphasizes Mendelian randomized analysis and provides poor details related to the primary objective of the study. It is not known from the method section how authors ensured that the data extracted were valid. It will be difficult for most readers to interpret the results of this novel method of analysis.
- **Response**: We are grateful for the reviewer's constructive feedback concerning the emphasis on Mendelian Randomization (MR) analysis and the perceived lack of detail regarding the study's primary objective and data validation within the methods section. In response to these concerns, we have undertaken several steps to enhance the clarity and comprehensiveness of our manuscript:

1. Clarification of Primary Objective: We have revised the introduction and methods sections to more explicitly state the primary objective of our study. This includes a clearer explanation of how our research aims to investigate the causal relationships between periodontal disease-related phenotypes (PDRP) and knee osteoarthritis (KOA) using MR analysis. By doing so, we hope to better align the readers' expectations with the study's goals from the outset.
2. Detailing Data Validation: In the revised methods section, we now provide a detailed account of the processes employed to ensure the validity of the GWAS data used in our MR analysis. This includes descriptions of the quality control measures taken, such as the selection of SNPs based on genome-wide significance thresholds, the assessment of SNP-exposure associations, and the use of robust instruments that meet the criteria for validity in MR studies[4,5]. We also elaborate on the use of sensitivity analyses, such as MR-Egger regression and the weighted median approach, to test for and address potential pleiotropy and other biases[6].
3. Enhancing Accessibility of MR Methodology: Acknowledging the reviewer's concern about the interpretability of MR analysis for readers unfamiliar with this approach, we have included a more accessible explanation of the MR methodology. This includes a simplified description of how genetic variants are used as instrumental variables to infer causality, the rationale behind this approach, and its advantages over traditional observational studies. Additionally, we provide references to foundational and educational resources on MR for readers seeking a deeper understanding.

We hope these revisions address the reviewer's concerns and make the manuscript more informative and accessible. Our intention is to ensure that the study's contributions to understanding the genetic underpinnings of the relationship between PDRP and KOA are clearly communicated and appreciated by a broad audience.

- **Comment:** It will be difficult for most readers to interpret the results of this novel method of analysis.
- **Response**: Mendelian Randomization (MR) studies have emerged as a pivotal tool in epidemiological research, offering several distinct advantages over traditional observational studies and circumventing some of the limitations inherent in randomized controlled trials (RCTs). The significance of MR studies, especially from a clinical perspective, is profound due to their higher evidence level compared to retrospective studies, the ability to infer causality without conducting RCTs, high credibility, low cost, and the potential to control for all confounding factors. Here’s a detailed explanation of these aspects:

1. **Higher Evidence Level Compared to Retrospective Studies**

MR studies leverage genetic variants as instrumental variables to estimate the causal effect of an exposure on an outcome. This approach is less susceptible to confounding and reverse causation, common issues in retrospective and even prospective observational studies. Since genetic variants are randomly assorted during meiosis, the allocation of these genetic instruments mimics the randomization process of an RCT, thus providing a natural experiment that can offer insights into causal relationships with a higher level of evidence than is typically achievable with observational study designs.

2. **No Need to Conduct RCTs**

RCTs are considered the gold standard for determining causality but are often expensive, time-consuming, and sometimes ethically or practically infeasible, especially for exposures that are difficult or impossible to manipulate directly (e.g., lifestyle factors or long-term dietary habits). MR studies can bypass these challenges by using existing genetic data, allowing researchers to infer causal relationships without the need for new RCTs.

3. **High Credibility**

The credibility of MR studies stems from their basis in genetic associations, which are less likely to be influenced by environmental confounders or biases that frequently affect observational studies. This genetic grounding helps ensure that the associations identified are more reflective of true causal relationships, lending a higher degree of credibility to the findings.

4. **Low Cost**

Since MR studies often utilize publicly available data from large-scale GWAS, they can be conducted at a fraction of the cost required for new clinical trials or large observational studies. This cost-effectiveness makes MR an attractive option for exploring causal hypotheses in epidemiology and public health research.

5. **Potential to Control for All Confounding Factors**

One of the most significant advantages of MR is its ability to provide estimates of causal effects that are free from confounding by environmental or lifestyle factors, assuming the selected genetic variants are valid instrumental variables. While no method can completely eliminate all forms of bias, MR is uniquely capable of controlling for confounders that are often unmeasured or unknown in traditional observational studies.

In summary, Mendelian Randomization studies offer a powerful, cost-effective, and credible approach to causal inference in epidemiology, bridging the gap between observational studies and RCTs. In recent years, the proliferation of Mendelian Randomization (MR) studies across prestigious journals has garnered widespread recognition within the scientific community, underscoring the method's substantial contribution to clinical research. This surge in MR-based publications not only highlights the growing acceptance of its findings but also emphasizes the pivotal role MR studies play in advancing our understanding of complex disease mechanisms and guiding evidence-based clinical interventions. Through bridging the gap between genetic epidemiology and clinical practice, MR studies continue to enrich the landscape of medical research, offering novel insights that drive the development of targeted therapies and inform public health policies.

**Discussion**

**Comment:** The discussion should summarize results, provide comparisons with existing literature, and consider the use of regression analysis.

**Response:** We appreciate the reviewer's insightful feedback and the suggestion to incorporate regression analysis for comparative purposes with existing literature. We have revised the Discussion section to begin with a clear summary of our key findings. We then discuss these results in the context of existing literature, highlighting both agreements and discrepancies. However, it is crucial to highlight a fundamental aspect of our study design that influences our methodological choices. Our analysis utilizes Mendelian Randomization (MR) to infer causal relationships between periodontal disease-related phenotypes (PDRP) and knee osteoarthritis (KOA), grounded in the use of single nucleotide polymorphisms (SNPs) data extracted from large, publicly available genome-wide association studies (GWAS). This approach integrates regression techniques within its framework, using genetic variants as instrumental variables to estimate the causal effect of exposure on outcomes. Unlike traditional regression analyses that require comprehensive clinical data, MR leverages genetic associations to control for confounding, providing robust causal inferences even in the absence of detailed clinical covariates. This methodological choice aligns with our study's objectives and the nature of our data, enabling us to explore causal links that conventional observational methods cannot adequately address.

**Conclusion**

We are committed to refining our manuscript to meet the high standards of the journal and the scientific community. We believe the revisions made in response to reviewer's comments have significantly enhanced the manuscript's clarity, robustness, and contribution to the field. Again, we thank you for their invaluable feedback and guidance.

**Response to Reviewer #2**

We sincerely appreciate the reviewer's positive assessment of our study and the recognition of its potential benefits for health workers in clinical practice. We are grateful for the recommendation for publication and acknowledge the concerns raised. Below, we provide a point-to-point response to address each of these issues:

- **Comment 1:** Selection bias related to study participants.
- **Response 1:**

Addressing concerns about selection bias related to study participants is crucial in epidemiological and genetic research. Selection bias occurs when the individuals included in the study are not representative of the target population, potentially leading to skewed or invalid findings. In the context of our Mendelian Randomization (MR) analysis, leveraging data from genome-wide association studies (GWAS), there are specific considerations and strategies we employed to mitigate the impact of selection bias:

1. **Broad Inclusion Criteria for GWAS Data**: The GWAS datasets utilized in our MR analysis were selected based on their broad inclusion criteria, aiming to encompass a wide range of individuals from the target population. This approach helps ensure that the genetic variants identified as instrumental variables (IVs) are relevant across a diverse subset of the population, reducing the risk of selection bias stemming from a narrow study sample.
2. **Analysis of Publicly Available Datasets**: By using publicly available GWAS data, our study benefits from the collective efforts of the research community to identify and catalog genetic associations across various populations. These datasets are often the result of large-scale studies designed to minimize selection bias through careful participant selection and data collection methods.
3. **Sensitivity Analyses to Assess Bias**: We conducted sensitivity analyses, including the MR-Egger regression, which provides an estimate of directional pleiotropy that might indicate bias, including selection bias. By examining the intercept term from the MR-Egger regression, we can assess whether there is evidence of bias that could influence the causal estimates.
4. **Consideration of Population Stratification**: While not selection bias per se, population stratification can lead to biased genetic associations if not properly accounted for. Our MR analysis incorporates data adjustments made in the original GWAS to control for population stratification, ensuring that the associations we observe are not confounded by ancestry-related differences within the study population.

Despite these measures, it's important to acknowledge that no study design is completely immune to selection bias. The populations represented in GWAS databases, while diverse, may still not capture the full spectrum of genetic and environmental variability present in the broader global population. This limitation underscores the need for cautious interpretation of our findings and the importance of validating these results in independent samples and diverse populations.

In conclusion, while we have taken several steps to mitigate the potential impact of selection bias in our MR study, we recognize the importance of ongoing vigilance and validation to ensure the generalizability and validity of our findings.

- **Comment 2:** The functional levels and dietary habits of the samples may influence the results which is not discussed.
- **Response 2**: We appreciate the reviewer's point regarding the potential influence of functional levels and dietary habits on our results. Due to the reliance on publicly available GWAS summary statistics for our Mendelian Randomization analysis, detailed individual-level data on lifestyle factors such as functional abilities and dietary patterns were not accessible. This limitation restricts our ability to directly assess or adjust for these potential confounders in our analysis. We acknowledge this as a constraint of the current study and highlight the importance of incorporating such lifestyle factors in future research to fully understand their impact on the relationship between periodontal disease-related phenotypes and knee osteoarthritis.
- **Comment 3:** Explain the significance of achieved Wald ratio in the discussion and its significance.
- **Response 3**:

In our Mendelian Randomization (MR) analysis, we employed the Inverse Variance Weighting (IVW) method to calculate the Wald ratio for each single nucleotide polymorphism (SNP). The Wald ratio, a critical component of MR analyses, is calculated by dividing the SNP-outcome association (the coefficient representing the effect of the SNP on the outcome) by the SNP-exposure association (the coefficient representing the effect of the SNP on the exposure). This ratio provides an estimate of the causal effect of the exposure (in this case, periodontal disease-related phenotypes, PDRP) on the outcome (knee osteoarthritis, KOA), under the assumption that each genetic variant used as an instrumental variable (IV) meets the necessary criteria for validity.

The IVW method aggregates these Wald ratios across multiple SNPs to provide a single, consistent estimate of the causal effect. This approach assumes that all instrumental variables are valid, meaning they are associated with the exposure, not associated with any confounders of the exposure-outcome relationship, and influence the outcome only through the exposure.

Our results, as summarized in the table, show the estimates of the causal effect of PDRP on KOA through different MR methods, including MR Egger, Weighted Median, IVW, Simple Mode, and Weighted Mode. Specifically, the Inverse Variance Weighted (IVW) method yielded an odds ratio (OR) of 3.85 with a standard error (SE) of 1.244 and a p-value of 0.279, indicating the estimated causal effect size along with its precision and statistical significance. The confidence interval (CI) from 0.34 to 44.05 further contextualizes this estimate within a range of plausible values.

While the IVW method provides a comprehensive estimate leveraging the strength of multiple SNPs, it's important to note the broad confidence intervals and nonsignificant p-values across our MR analyses, including the IVW. These findings suggest a degree of uncertainty around the estimated causal effect, warranting cautious interpretation. Such outcomes underline the complexity of disentangling the genetic underpinnings of the relationship between PDRP and KOA and emphasize the need for further research to validate these findings and explore the mechanisms at play.

In conclusion, the Wald ratio serves as a cornerstone in our MR analysis, enabling the estimation of causal effects by leveraging genetic variation as a proxy for the exposure of interest. This methodological choice, coupled with the comprehensive approach of the IVW method, offers valuable insights into the potential causal relationship between PDRP and KOA, contributing to our understanding of these complex conditions.

- **Comment 4:** Q statistic did not show significant difference, discuss in detail the effect of q and p values achieved. (Q = 2.945; P = 0.567).
- **Response 4:**

In response to the query regarding the Q statistic and its implications for our analysis, we appreciate the opportunity to elaborate on these aspects further. The Cochran's Q test is a statistical measure used to assess heterogeneity across instrumental variables (IVs) in meta-analyses, including Mendelian Randomization (MR) studies like ours. For our analysis, the Q statistic was calculated to be 2.945 with a corresponding p-value of 0.567.

According to the standard criteria we adopted, significant heterogeneity among the IVs is indicated by a p-value less than 0.05. In our case, the p-value of 0.567 suggests that there is no significant heterogeneity among the instrumental variables used in our analysis. This result supports the assumption that the effect sizes estimated by the different IVs are consistent with each other across the various genetic variants considered. Consequently, this allowed us to proceed with a fixed-effects Inverse Variance Weighting (IVW) model for our primary analysis, as opposed to employing a random-effects model, which would be more appropriate in the presence of significant heterogeneity.

The absence of significant heterogeneity, as indicated by the Q statistic, strengthens the validity of our findings by suggesting that the IVs selected for our MR analysis share a common effect size in their relationship with both the exposure (periodontal disease-related phenotypes, PDRP) and the outcome (knee osteoarthritis, KOA). This homogeneity among IVs is crucial for the reliability of MR analyses, as it underlines the consistency of the causal inferences drawn from our study.

However, it's important to acknowledge that the absence of detected heterogeneity does not entirely rule out its existence. The power to detect heterogeneity depends on the number of IVs and the sample size. Therefore, while our findings suggest a homogeneous effect among the selected IVs, we remain cautious in our interpretation, understanding that MR analyses, like all statistical methods, have their limitations.

In summary, the results from the Cochran's Q test in our study provide reassurance regarding the consistency of our instrumental variables, supporting the use of a fixed-effects IVW approach. This contributes to the robustness of our conclusions regarding the causal relationship between PDRP and KOA. Nonetheless, we continue to advocate for further research to confirm these findings and explore the complexities of these relationships in greater detail.

**Conclusion**

In closing, we are deeply thankful for the insightful feedback provided by the reviewer. We believe that addressing these concerns has not only clarified important aspects of our study but also highlighted areas for future research. We hope our responses adequately address the issues raised and further substantiate the value of our contributions to the field.\

References:

[1] Dudbridge, F. (2013). Power and Predictive Accuracy of Polygenic Risk Scores. PLoS Genetics, 9(3), e1003348.

[2] Evangelou, E., & Ioannidis, J. P. (2013). Meta-analysis methods for genome-wide association studies and beyond. Nature Reviews Genetics, 14(6), 379–389.

[3] Davey Smith, G., & Hemani, G. (2014). Mendelian randomization: genetic anchors for causal inference in epidemiological studies. Human Molecular Genetics, 23(R1), R89–R98.

[4] Hemani G, Bowden J, Davey Smith G. Evaluating the potential role of pleiotropy in Mendelian randomization studies[J]. Human molecular genetics, 2018, 27(R2): R195-R208.

[5] Boehm F J, Zhou X. Statistical methods for Mendelian randomization in genome-wide association studies: a review[J]. Computational and structural biotechnology journal, 2022, 20: 2338-2351.

[6] Burgess S, Thompson S G. Interpreting findings from Mendelian randomization using the MR-Egger method[J]. European journal of epidemiology, 2017, 32: 377-389.
